# Long-Term Radial Artery Grafts with Previous Midterm Proven Patency

**DOI:** 10.21470/1678-9741-2021-0074

**Published:** 2023

**Authors:** Roberto Rocha e Silva, Maxim Goncharov, Fabiane Letícia de Freitas, Omar Asdrúbal Vilca Mejia

**Affiliations:** 1 Department of Cardiovascular Surgery, Instituto do Coração, Universidade de São Paulo, São Paulo, São Paulo, Brazil

**Keywords:** Constriction, Pathologic, Coronary Artery Bypass, Coronary Artery Disease, Mammary Arteries, Multidetector Computed Tomography.

## Abstract

**Introduction:**

Left internal thoracic artery to left anterior descending artery (LITA-LADA) grafting has become a fundamental part of coronary artery bypass grafting (CABG). This grafting has led to an increased use of other arterial conduits, of which the radial artery (RA) is the most popular. Whether RA can have the same long-term patency as LITA is controversial. The objective of this study is to access the long-term clinical follow-up and, when available, the patency rate of RA grafts.

**Methods:**

Twenty-six patients from a previous study with critical stenosis in all target vessels underwent complete arterial CABG with LITA and RA grafts from 1996 to 2003. They all underwent midterm multidetector computed tomography after surgery with the association of at least one patent LITA and one patent RA graft.

**Results:**

Twelve patients (46%) are alive with no angina symptoms. Six patients underwent a second image exam 12 to 16 years (average of 14 years) after surgery, with a total of six LITA-LADA and 14 RA grafts with 100% patency rate. Clinical follow-up five to 23 years after surgery (average of 14 years) showed only one death 12 years after surgery related to coronary artery disease (CAD) (3,8%). Another 12 patients died of non-CAD.

**Conclusion:**

Patients with midterm associated LITA and RA patent grafts show similar optimal long-term patency rates of both types of grafts with excellent clinical outcome.

## INTRODUCTION

The left internal thoracic artery (LITA) is the conduit of choice in coronary artery bypass grafting (CABG) because of its superior graft patency, reduced cardiac events, and enhanced short and long-term survival^[^1^]^. In the search for other conduits for total arterial revascularization, Acar^[^2^]^ reintroduced the radial artery (RA) graft after a long-term observation of patent RA conduits that were thought to have been occluded in the early postoperative period.

Other studies^[^3^-^6^]^ demonstrated complete arterial revascularization compared to conventional use of saphenous vein conduits, with long-term survival and intervention-free survival.

Schwann reported^[^7^]^ that RA and right internal thoracic artery (RITA) have similar long-term results, which are superior to the saphenous vein grafts (SVG) used.

We have previously reported the immediate follow-up study on complete arterial revascularization with LITA and RA in elective *versus* emergency surgery presenting similar clinical outcomes for both groups^[^8^]^.

Also, we have reported the midterm follow-up of this same group. Thirty patients (71% of survivors) were studied by 64-slice multidetector computed tomography (MDCT) with overall patency rate for RA of 83% (53/64). Thus 26 patients (87%) had the association of at least one LITA and one RA patent graft^[^9^]^.

The objective of this study is to access the long-term clinical follow-up and, when available, the patency rate of the LITA and RA grafts of the 26 patients submitted to complete arterial revascularization with LITA and RA enrolled in our previous study. They all have midterm proven association of at least one patent LITA and one patent RA graft by MDCT.

## METHODS

The patients were selected from our previous study^[^9^]^ from 2007. All patients had critical stenosis (> 70%) in all target vessels, and the surgeries were performed from 1996 to 2003 by the same surgeon. They all had complete arterial revascularization with LITA and RA (without any other type of graft). Forty-seven patients underwent CABG. Most of the patients were men (79%), between 50 and 60 years, and had triple-vessel coronary artery disease (81%). There was no preoperative use of an intra-aortic balloon pump. Of these, 30 patients were studied by MDCT for 51.7±19.8 months after surgery. A total of 36 LITA and 64 RA grafts were studied. The RA grafts patency rates were 53/64 (83%). The association of at least one patent LITA and one patent RA graft was present in 26 patients (87%), which were selected for this study.

The surgical approach of the previous study was through sternotomy with cardiopulmonary bypass. After March 2003, the off-pump surgery was performed in 11 out of 19 cases (57%). RA was harvested with a no-touch technique and with no mechanical expansion before implantation^[^10^]^. After harvesting, the graft was left *in situ* with topic papaverine until the initialization of coronary grafting. LITA was harvested in all but one case (a reoperation with patent LITA-left anterior descending artery [LADA] graft). RA was grafted to all target vessels, but in one patient it was grafted to LADA. In this case, LITA was grafted to RA, which was grafted as an arch passing through the distal LADA, proximal LADA, and diagonal and marginal branches. LITA was grafted to LADA and, when needed and possible, to the diagonal branch. Each distal anastomosis was considered an independent graft. RA proximal anastomoses were done in a “Y” shape with LITA, except when LITA was subjectively evaluated to have a small caliber. Of these 47 patients, five died from non-cardiac complications, and 12 were lost to follow-up. Consequently, 30 patients (71% of survivors) were studied by MDCT.

MDCT was performed without complications 51.7±19.8 months after surgery. The RA patency rate was 83% (53/64). The LITA patency rate was 89% (32/36). Twenty-six patients had the association of at least one patent LITA and one patent RA graft. These 26 patients were selected for our long-term study.

Medical records were reviewed, and, in the absence of recent data (> 1 year), telephone contact was made. The data analyzed was available graft image and clinical outcome.

This study represents the final follow-up of patients presented in previous studies^[^8^,^9^]^ approved by the ethics committee under number 5605276. The ethics committee waived the requirement to informed consent due to the retrospective nature of the study.

## RESULTS

Of the 26 patients from the previous study, 12 (46%) were alive during the current follow-up, 13 (50%) died, and one patient was not found and was excluded.

Alive patients had a mean of 18 years (standard deviation [SD]±1,85) between surgery and a second follow-up. Four of them could perform a second image exam. Only one had undergone angioplasty during this period in the right coronary artery that has not been approached during the first surgery.

Patients who have not survived until the second follow-up had a mean of 11,3 years (SD±4,1, min. 4,8, max. 17,0) of survival after surgery. The main reason for death was cancer and its complications in six patients (46%). One patient (7%) had sudden death. One patient (7%) had a lethal head trauma. The remaining five patients (38%) had unspecified cause of death. One patient had undergone one more cardiovascular surgery (aortic aneurysm) between the first surgery and death.

In total, a second image exam was performed in six patients 12 to 16 years (average of 14 years, SD±1,75) after surgery. To compare the results there was no need to use any statistical approach, because all six LITA and all 14 RA grafts were patent (100%). Between the first and the second follow-up image exam, two patients died (13 and 15 years after surgery) of unspecified cause and cancer, respectively.

Thus, 26 patients aged from 65 to 88 years (average of 75 years old) were followed up clinically after surgery for five to 23 years (average 14 years). It showed that of all patients, 12 people (46%) were still alive. Only one death (3.8%) 12 years after surgery is associated with coronary artery disease. In the period from six to 15 years after surgery (average 12 years), six patients died of cancer, one person died from injuries, and five people died of unknown causes.

## DISCUSSION

Our results with no mortality compared to literature data^[^11^,^12^]^ suggest that our total arterial graft approach is safe.

The superiority of LITA as a graft for CABG is beyond dispute, but there is controversy over the kind of graft that should be associated with LITA.

Our previous study demonstrated excellent midterm patency rates for RA, both in the elective and the non-elective scenarios. Tatoulis^[^13^]^ reported higher levels of RA patency rates associated with more severe coronary stenosis. Because all patients in our study had critical stenosis (> 70%) in all target vessels, we found optimal RA graft patency rates in our long-term follow-up.

Miana compared RA and RITA as a second graft, demonstrating similar immediate results, but with a longer operative time in the RITA group^[^14^]^. In our previous studies, there was no time measurement for RA harvesting, but it was always harvested simultaneously with the sternum opening; it was always ready before the LITA harvesting was finished, thus wasting no time.

There is no consensus in literature^[^15^]^ if the location of the target vessel has an impact on RA patency rate. Our study had similar excellent patency rates for all target vessels. The use of RA sequential anastomosis has the same patency rates as the single vessel graft, but it can provide complete arterial revascularization with fewer grafts and therefore reduces harvesting time and the number of proximal anastomoses. Moreover, this will shorten total surgical time.

Regarding clinical aspects, the progression of atherosclerosis in followed patients was very low. Only one patient died because of cardiac reasons that might be caused by atherosclerosis, and one patient had angioplasty of an artery that has not been grafted. Most of the patients were asymptomatic during all follow-up. That fact can be explained by recent literature. Dimitrova et al.^[^16^]^ compared cumulative graft patency and disease progression in arterial (RA) and venous (SVG) grafts. This study showed that disease progression in grafted native coronary arteries to the lateral territory with a patent RA graft was 11% *versus* 50% with a patent SVG. That explains the slow progression of an atherosclerosis in our group of patients.

Patients included in this report had proven midterm association of at least one patent LITA and one RA graft by MDCT. They also had excellent midterm clinical evolution with most patients being asymptomatic and free from reintervention. In patients who underwent a second image exam, all grafts were pervious. As expected, the long-term clinical evolution was excellent with most patients being asymptomatic and free from reintervention. This technique has long-term benefits because RA, which has medium-term patency, appears to have similar endurance to LITA.

### Limitations

There are some limitations to the present study that must be recognized. First, this study was not performed with a control group receiving a saphenous vein that would be used in comparison to the RA. Second, this study had a small sample size. Third, we had 10 late deaths and a 19,2% loss of surviving patients, thus this combination might have overestimated the patency rates by selecting for patients who survived and were available for the study.

## CONCLUSION

In our long-term follow-up observational study, patients with midterm associated LITA and RA patent grafts show similar optimal long-term patency rates of both types of grafts with excellent clinical outcome.
